# Genomics and pharmacogenomics of sepsis: so close and yet so far

**DOI:** 10.1186/s13054-016-1374-6

**Published:** 2016-07-07

**Authors:** James A. Russell

**Affiliations:** Centre for Heart Lung Innovation, St. Paul’s Hospital, 1081 Burrard Street, Vancouver, BC V6Z 1Y6 Canada; Division of Critical Care Medicine, St. Paul’s Hospital, University of British Columbia, 1081 Burrard Street, Vancouver, BC V6Z 1Y6 Canada

## Abstract

Sapru et al. show in this issue of *Critical Care* that variants of thrombomodulin and the endothelial protein C receptor, but not protein C, are associated with mortality and organ dysfunction (ventilation-free and organ failure-free days) in ARDS. Hundreds of gene variants have been found prognostic in sepsis. However, none of these prognostic genomic biomarkers are used clinically. Predictive biomarker discovery (pharmacogenomics) usually follows a candidate gene approach, utilizing knowledge of drug pathways. Pharmacogenomics could be applied to enhance efficacy and safety of drugs used for treatment of sepsis (e.g., norepinephrine, epinephrine, vasopressin, and corticosteroids). Pharmacogenomics can enhance drug development in sepsis, which is very important because there is no approved drug for sepsis. Pharmacogenomics biomarkers must pass three milestones: scientific, regulatory, and commercial. Huge challenges remain but great opportunities for pharmacogenomics of sepsis are on the horizon.

This issue of *Critical Care* presents a novel human genomics study showing that variants of thrombomodulin (TM) and the endothelial protein C receptor (EPCR), but not protein C, are associated with mortality and organ dysfunction (ventilation-free and organ failure-free days) in ARDS—that is, they are prognostic biomarkers [1]. Strengths include the cohort, gene, and variant selections, Hardy–Weinberg equilibrium, correlation of variants with plasma protein levels, correction for multiple comparisons, a haplotype model, a multivariant approach, and a priori sample size calculation. A relative weakness was the reliance on literature for biological plausibility of the “significant” variants. These innovative insights could lead to “predictive biomarkers” for response to recombinant human TM and even activated protein C (APC) in sepsis.

Genomics and pharmacogenomics (PGx) are pivotal to fields such as cancer and cardiovascular medicine. In cancer, PGx biomarker(s) include trastuzumab (Herceptin, HER2), irenotecan (UGT1A1*28), azothioprine and 6-mercaptopurine (TPMT), capecitabine (dihydropyrimidine dehydrogenase), and cetuximab/panitumumab (KRAS)—these drugs are very frequently given according to the specific PGx biomarker. In cardiovascular medicine, clopidogrel (CYP2C19) and warfarin (VKORC1) are well-documented PGx biomarkers that indicate altered efficacy and safety respectively. PGx biomarkers are used increasingly in clinical practice.

Sepsis has gone through 15 years of discovery of many genomic biomarkers [2, 3]. A PubMed search for “sepsis and polymorphism” yields 1199 publications. Let us define some terminology: a prognostic biomarker identifies prognosis (e.g., increased risk of death); a diagnostic biomarker diagnoses condition (e.g., sepsis diagnostic); and a predictive biomarker (companion diagnostic) uses genomics to define response to a drug (see http://www.fda.gov/MedicalDevices/ProductsandMedicalProcedures/InVitroDiagnostics/ucm407297.htm). Over 100 drugs have approved predictive biomarkers on the drug label (http://www.fda.gov/drugs/scienceresearch/researchareas/pharmacogenetics/ucm083378.htm)!

About one quarter of the human genome changes expression in sepsis [4], so it is not surprising that hundreds of variants are described in sepsis [3]. However, none of these prognostic genomic biomarkers are used clinically, probably because of lack of clinical utility (i.e., the test result would not change a clinician’s behavior). Nonetheless, genomics of sepsis studies have identified key pathways associated with specific organ dysfunctions and mortality, and have identified drug targets in sepsis (e.g., proprotein convertase subtilisin/kexin type 9 (PCSK9) [5]). Variants of PCSK9 were associated with outcomes of sepsis, and post treatment of cecal ligation and perforation model mice with PCSK9 inhibitors decreased inflammation, cardiovascular dysfunction, and mortality; thus, PCSK9 inhibition could be effective in sepsis [5]. This example could be expanded to other genes and novel drugs.

Predictive biomarker discovery often follows a candidate gene approach, utilizing knowledge of drug receptors, transporters, enzymes that metabolize a drug, and drug target pathways. Predictive biomarkers often have high clinical utility. FDA-approved drug labels have a hierarchy of recommendations for companion diagnostics: (1) for information (i.e., descriptions of published studies of PGx related to the drug); (2) recommended—physicians are encouraged to measure the biomarker; and (3) required—physicians MUST use the companion diagnostic to prescribe the drug. The required companion diagnostic relates to trastuzumab: HER2 must test positive for identifying good responders to order trastuzumab.

The PGx biomarker discovery pathway is arduous, time-consuming, and expensive. A successful PGx biomarker must pass three milestones: scientific, regulatory, and commercial. Scientific steps include: a decision regarding a nonhypothesis-driven (genome-wide) vs a candidate gene approach, RCTs, significant drug/PGx biomarker interaction, validation often in a separate RCT, and validation of a rapid turnaround time (TAT) kit in real time. The regulatory node includes many submissions and visits to regulators before and after each study. Regulators have approved PGx biomarkers in cancer that were assessed at the end of pivotal RCTs provided that the biomarker hypothesis was logged before locking the RCT dataset. Thus, selection of PGx biomarkers may occur in parallel with RCT execution. Finally, the commercial node includes costs of kits, reimbursement methods and amounts, FDA-approved and EMEA-approved manufacturing, and distribution of a rapid TAT kit (and sometimes a unique “box” for measuring the biomarker) to hospitals (laboratories and/or ICUs or EDs).

PGx could be applied to enhance efficacy and safety of drugs in use for sepsis and septic shock including norepinephrine, epinephrine, vasopressin, and corticosteroids (CS) (Table 1); known genomic variants intersect with these drugs. Genomics of the CS and vasopressin (*AVP*) axes have been well studied for prediction of response to CS (and less so vasopressin), because CS and *AVP* variants are widely studied in many conditions and because CS are used in so many conditions (Table 1).

**Table 1:** Potential pharmacogenomic biomarkers and steps to discovery for drugs used clinically, drugs in development, and drugs that could be resurrected in sepsis

Drugs in use clinically in sepsis and septic shock	Potential pharmacogenomic biomarkers	First step(s) to discovery/validation of PGx biomarkers
Norepinephrine (NE), epinephrine (EP), dobutamine	ARs: ADBR1 and ADBR2 [7] SNPs ADRA1A and B SNPs G-protein subunits (α, β, and γ) of ADR2a α2A N251K and α2C Δ322-325 (common AR SNPs) ADRA2A, B and C SNPs ADCY9 SNPs	Genotyping of patients in RCT (e.g., Annane et al. [8])
Vasopressin	LNPEP SNPs [9], AVPR1a, AVPR1b, and AVPR2 SNPs	Genotyping of patients in RCT (e.g., Russell et al. [10])
Corticosteroids	CRF SNPs GRs [11] GR heterocomplex gene STIP1 SNPs: ER22/23EK (common GR SNP) GR: N363S (common GR SNP) GR: 9β A/G (common GR SNP) Bcll (common GR SNP) GLCC1 SNPs ABCB1 SNPs IL-1β SNPs NR3C1 SNPs MR SNPs (e.g., TthIIII, MRI180V, and MR-2G/C) NALP1 SNPs NK2R SNPs CTLA4 SNPs	Genotyping of patients in RCT (e.g., Sprung et al. [12])
Examples of drugs used in sepsis that have FDA-approved companion diagnostics		
Diazepam	CYP2C19 (poor metabolizers of diazepam)	FDA label^a^
Methylene blue	G6PD deficiency	FDA label^a^
Omeprazole, pantoprazole	CYP2C19 (poor metabolizers of Omeprazole and pantoprazole)	FDA label^a^
Resperidone	CYP2D6 (poor metabolizers of respiradone)	FDA label^a^
Drugs in or near development		
Thrombomodulin (TM)	TM, EPCR [1], and	Genotyping of patients in RCT^b^
	PROC SNPs	
Selepressin	LNPEP SNPs [9], AVPR1a SNPs	Genotyping of patients in RCT^b^
Angiotensin II (ANG II)	AGT SNPs AGTRAP SNPs [13] ACE SNPs and ID AT1R and 2R SNPs	Genotyping of patients in RCT^b^
PCSK9 inhibitor	PCSK9 SNPs [5]	Genotyping of patients in RCT^b^
IL-7	IL-7, IL-7ra SNPs	Genotyping of patients in RCT^b^
Esmolol	ADBR1A and B SNPs [7]	Genotyping of patients in RCT
Resurrecting a drug		
Activated protein C	TM, EPCR [1] PROC SNPs [14]	Genotyping of patients in RCT (e.g., Ranieri et al. [15])

*PGx* pharmacogenomics, *SNP* single nucleotide polymorphism, *RCT* randomized controlled trial, *AR* adrenergic receptor

*ADRA* adrenergic alpha receptor, *ADBR1/*2 β1/2-adrenergic receptor, *LNPEP* leucyl/cystinyl aminopeptidase (vasopressinase), *AVPR1a*/*b* gene name for V1a/b receptors, *CRF* corticotropin-releasing factor, *ADCY9* adenylyl cyclase type 9, *EPCR* endothelial protein C receptor, *PROC* protein C, *GR* membrane-bound and cytosolic glucocorticoid receptor, *GLCCI1* glucocorticoid-induced transcript 1 gene, *ABCB1* gene codes for P-glycoprotein, *NR3C1* glucocorticoid receptor gene, *MR* mineralocorticoid receptor, *NALP1* NACHT leucine-rich-repeat protein 1, *NK2R* neurokinin receptor 2, *CTLA4* anti-cytotoxic T lymphocyte-associated antigen-4, *PCSK9* proprotein convertase subtilisin/kexin type 9, *CYP2C19* cytochrome P450 2C19, *G6PD* glucose-6-phosphate dehydrogenase, *AGT* angiotensinogen gene, *AGTRAP* angiotensin II receptor-associated protein, *ACE* angiotensin-converting enzyme, *ID* insertion/deletion polymorphisms, *AT1/ 2R* angiotensin-II type 1 and 2 receptor gene, *IL-7ra* interleukin-7 receptor alpha chain

^a^These markers are already approved for clinical use with shown drugs on the drug FDA label. No further RCTs are required for “on label” clinical use of the companion diagnostic strategy in practice

^b^Overviews of RCTs of: thrombomodulin (https://clinicaltrials.gov/ct2/show/NCT01598831?term=thrombomodulin+in+sepsis&rank=2), selepressin (https://clinicaltrials.gov/ct2/show/NCT02508649?term=selepressin+in+shock&rank=1)

angiotensin II, (https://clinicaltrials.gov/ct2/show/NCT01393782?term=angiotensin+II+in+septic+shock&rank=2), and IL-7 (https://clinicaltrials.gov/ct2/show/NCT02640807?term=il-7+in+sepsis&rank=1)

PGx can also enhance drug development, very important since there is no approved drug for sepsis. PGx could increase chances of drug development success in sepsis; that is, precision medicine to enrich the heterogeneous sepsis cohorts [2, 6]. Potential predictive biomarkers/companion diagnostics could be used with recombinant human TM, selepressin, angiotensin II, PCSK9 inhibitor, IL-7, and esmolol (Table 1). Studies of PGx of ACE inhibitors in cardiovascular disease and IL-7 in cancer could inform angiotensin II and IL-7 PGx in sepsis (Table 1). Several drugs used clinically in sepsis have proven companion diagnostics (Table 1).

Finally, PGx could resurrect “dead” drugs by increasing efficacy. APC could be resurrected by using genetic variants such as those discovered by Sapru et al. [1] that might mark patients who have an enhanced response to APC to enrich patient selection in a future RCT (Table 1).

In summary, there remain huge challenges but great opportunities for genomics, and I think more importantly for PGx of sepsis. We are close—but yet so far because there are many complex steps and milestones to bring a novel PGx biomarker to septic patients and their caregivers. I remain very optimistic that researchers such as Sapru et al. [1] and other scientists in the field are up to the challenge!

## Abbreviations

APC, activated protein C; CS, corticosteroids; EPCR, endothelial protein C receptor; PCSK9, proprotein convertase subtilisin/kexin type 9; PGx, pharmacogenomics; RCT, randomized controlled trial; TAT, turnaround time; TM, thrombomodulin
